# Sustainable livestock systems to improve human health, nutrition, and economic status

**DOI:** 10.1093/af/vfz041

**Published:** 2019-09-28

**Authors:** Padmakumar Varijakshapanicker, Sarah Mckune, Laurie Miller, Saskia Hendrickx, Mulubrhan Balehegn, Geoffrey E Dahl, Adegbola T Adesogan

**Affiliations:** 1 International Livestock Research Institute, Hyderabad, India; 2 Department for Public Health and Health Professions, University of Florida, Gainesville, FL; 3 Feed the Future Innovation Lab for Livestock Systems, Institute of Food and Agricultural Sciences, University of Florida, Gainesville, FL; 4 School of Medicine, Friedman School of Nutrition Science and Policy, and Eliot-Pearson Department of Child Study and Human Development, Tufts University, Boston, MA; 5 Department of Animal Sciences, Institute of Food and Agricultural Sciences, University of Florida, Gainesville, FL; 6 Department of Animal, Rangeland and Wildlife Sciences, Mekelle University, Mekelle, Tigray, Ethiopia

**Keywords:** green house gas emission, health, livestock, sustainability

**Implications**

- Sustainable livestock systems contribute to food security, economic and environmental stewardship, and sociocultural needs and are vital for achieving most of the United Nation’s Sustainable Development Goals.
- Livestock production contributes to sustainability through use of uncultivable land for food production, conversion of energy and protein sources that cannot be used by humans into highly nutritious animal-sourced food and reduction of environmental pollution with agroindustrial by-products, while generating income and supporting livelihoods of millions of people all over the world.
- Some livestock systems are particularly effective at carbon sequestration and hence reducing greenhouse gas emissions that contribute to global warming.
- Livestock production offers the greatest potential to reduce greenhouse gas emissions from agriculture and animal scientists have devised several effective strategies that can reduce such emissions from livestock systems by up to 30%.
- Most of the current discourse on sustainability focuses on one albeit important factor—the environment. Equally important factors are the need to ensure food and nutritional security for the growing global population in a culturally acceptable manner that ensures its accessibility, affordability, and safety.
- While livestock systems generally contribute to sustainability, poorly managed livestock systems may have adverse effects on the environment and human and animal health and welfare.

## Introduction

The most common words associated with sustainability are “environment,” “social,” and “economic.” Thus, sustainability is a holistic concept that jointly considers ecological, social, and economic dimensions of a system or intervention for long-lasting prosperity. Experience shows that economic development at the cost of ecology does not last; therefore, it is critical to harmonize ecology with development. This also applies to livestock systems, which should be economically viable for farmers, environmentally friendly or at least neutral, and socially acceptable in order to be considered sustainable.

There are different types of livestock production systems, depending on availability of resources, environmental conditions, and social and economic contexts, and they vary considerably in sustainability. These livestock systems include the grassland-based extensive systems, intensive landless systems, and mixed farming systems among others. These systems contribute significantly to human nutrition and livelihoods and provide important ecosystem services. However, if not properly managed, they can also cause nutrient and environmental pollution and land degradation.

With increasing global awareness about climate change and studies indicating that livestock is one of the contributors to greenhouse gases, environmental degradation, and loss of biodiversity, various concerted efforts have been aimed at developing and or ensuring the sustainability of livestock systems that deliver economic and ecosystems services without compromising the future integrity, health, and welfare of the environment, humans, and animals. Increasing competition for the requisite resources for feed and food production, especially under more intensive livestock production systems, has raised concerns about the economic and environmental sustainability of some livestock production systems. Feed production and processing, and enteric fermentation of feed contribute to 45% and 39%, respectively, of the total emissions from agriculture (Steinfeld et al., 2006). About 90% of livestock emissions are produced by ruminants through enteric fermentation (188 million tons) and the remaining 10% from manure (Swamy and Bhattacharya, 2006). In addition, inadequately managed livestock production systems may cause negative environmental consequences such as eutrophication in intensive high input systems, overgrazing, and soil and rangeland degradation in extensive systems and negative human health outcomes.

Even though inadequately managed livestock systems may have adverse effects on the environment, widely quoted statistics about their contribution are misleading. Most do not reflect the diversity of livestock production systems nor differences between production systems dominant in various countries even for a given species. For instance, an often-cited statistic is that livestock contribute 18% of greenhouse gases globally (Steinfeld et al., 2006), more than that for the transportation industry, but that analysis is incorrect and has been corrected by the authors (Mottet and Steinfeld, 2018). Moreover, interventions can help reduce the carbon footprint of livestock production, while improving productivity. For example, with improved management and feeding strategies, the carbon footprint per billion kilograms of beef produced in 2007 was reduced by 16.3% compared with equivalent beef production in 1977 (Capper, 2011).

**Figure 1.**
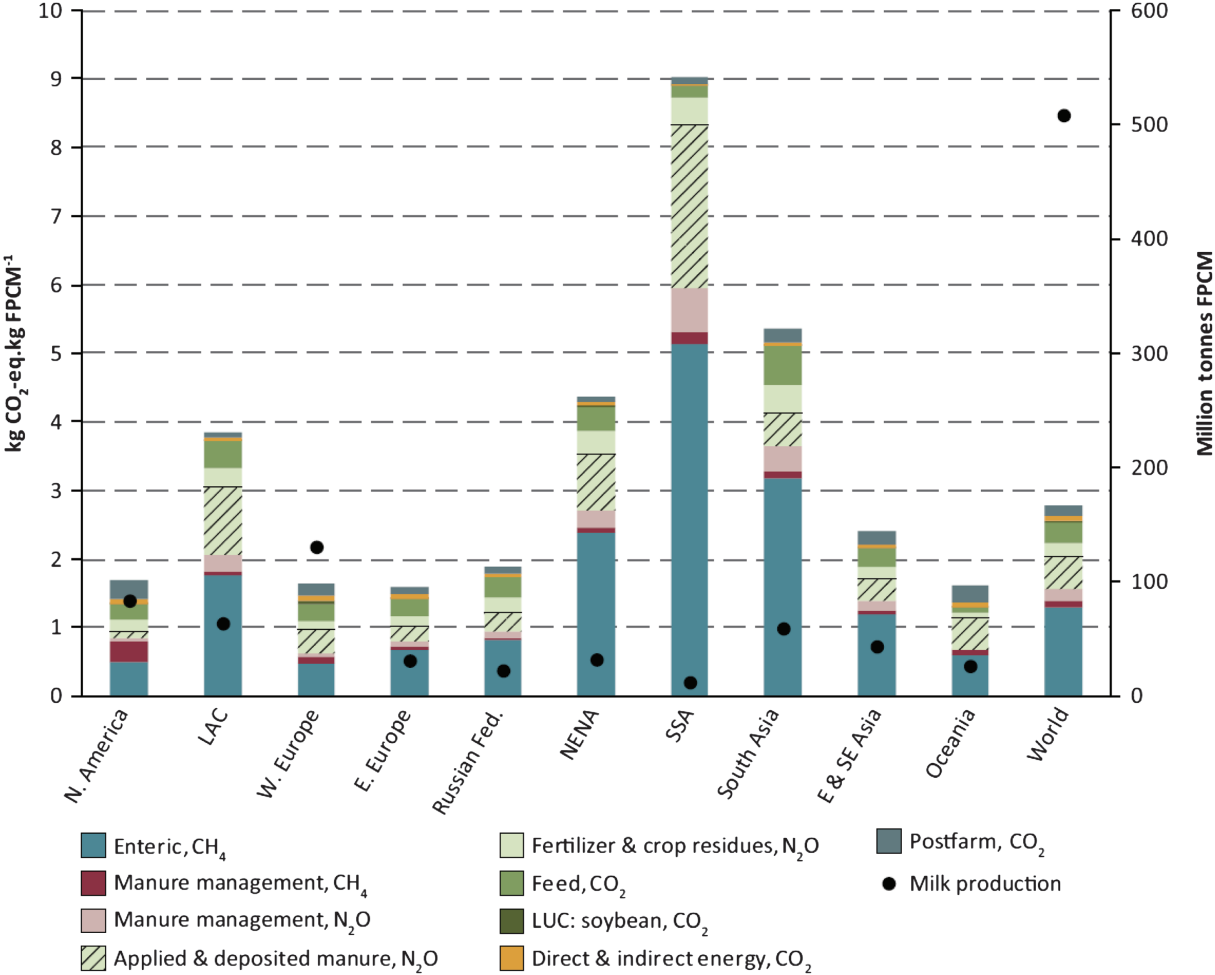
Regional variation in greenhouse gas emission intensities. Reprinted with permission from “Tackling climate change through livestock—A global assessment of emissions and mitigation opportunities” (Gerber et al., 2013).

When comparing greenhouse gas emissions of various livestock production systems, it is critical to take the need for environmental stewardship as well as food security into account to ensure the sustainability of the system. An index which takes both into account is the emissions intensity measure, which relates greenhouse gas emissions to food produced by the system. This important index shows that methane production per unit of food produced in several low- and middle-income countries is much greater than in some developed countries (Figure 1). This does not imply that the production systems in the developed countries should be copied entirely by low- and middle-income countries; rather, each country should evaluate and implement the aspects of developed country production systems that will sustainably intensify their production systems and thereby increase food production while reducing greenhouse gas emissions.

Often ignored is the fact that livestock systems contribute to global sustainability by providing various ecosystem services. For instance, a recent meta-analysis of 86 studies that examined various agroforestry systems revealed that net accumulation of soil carbon or sink of greenhouse gases was greatest when grassland was converted to silvo-pastures combining trees, forage, and livestock (Feliciano et al., 2018). Land maintained for livestock grazing has lower greenhouse gas emission than the same land converted for crop production. Rates of soil loss in U.S. croplands are more than four times that of grazing lands. Grazing lands sequester more carbon per unit area compared with cultivated croplands (Diaz et al., 2012). Furthermore, globally more than half (57%) of the 2.5 billion hectares of land used for producing forage is unsuitable for food production (Mottet et al., 2017). Thus, forage crops make productive use of noncultivable land. In addition, since only 14% of the feed consumed by livestock is edible by humans, the remaining 86%, including by-products, crop residues, and grasses or fodder, is converted into human food contributing to incomes and avoiding environmental pollution from burning or dumping the residues and by-products (Mottet et al., 2017). Even when livestock consume human-edible proteins, their net protein contribution is positive. For example, in U.S. beef production systems, the ratio of human-edible protein in animal-source foods to that in animal feed is always greater than one (Baber et al., 2018). Thus, livestock are net contributors to human protein requirements (Baber et al., 2018) and in fact livestock contribute to 13% and 28% of the global protein and energy, respectively (FAO, 2009).

Animal scientists have developed nutritional, genetic, health, and management strategies to reduce greenhouse gas emission intensities by as much as 30% (Gerber et al., 2013). Indeed, the concept of sustainable diets that are profitable, ethically and socio-culturally acceptable, and environmentally benign is emerging as one of the key solutions to ensuring the sustainability of livestock production systems. Considering the competition between feed and food systems, the concept of sustainable diets stipulates that future feed systems should focus on increased efficiency of conversion of fibrous feeds such as crop residues with high content of poorly digestible structural carbohydrates (lignin and cellulose) into human-consumable animal products. Sustainable diets and feed systems, therefore, have a potential for maintaining profitability of feed systems while reducing their negative environmental and social impacts (Bocquier and González-García, 2010). The adoption of such sustainable animal diets will require multidisciplinary input into the development of objective indicators. Future research into sustainable livestock diets should target both animal physiology and farmers’ practices to develop an integral, dynamic, and flexible conceptual perspective (Bocquier and González-García, 2010).

## Sustainable Livestock Production for Human Nutrition

Assessment of sustainability of livestock food systems usually focuses on GHG emissions from the foods produced. However, this approach does not account for the nutritional, health, and other benefits livestock provide in various production systems. These benefits offset the greenhouse gas they produce, which are declining because of the introduction of improved livestock management systems (Capper, 2011). The larger carbon footprint generated by livestock compared with other food sources are necessary trade-offs because livestock systems provide nutrient-rich products that are vital for health and wellbeing (White and Hall, 2018).

### Human nutrition, malnutrition, and stunting

The nutrient requirements of human beings include macronutrients (carbohydrates, protein, and fat) and micronutrients (vitamins and minerals). Malnutrition is defined as a deficiency, excess, or imbalance in nutrient intake versus nutrient requirements. Both undernutrition and overnutrition may have serious consequences. Undernutrition during infancy and childhood is widespread in low- and middle-income countries and is most commonly classified as stunting (low height-for-age) or wasting (low weight-for-height). Stunting usually reflects chronic malnutrition and frequent infections while wasting indicates acute significant food shortages and/or diseased status, and is a strong predictor of mortality. About 1 in 5 or 151 million children in the world are stunted, and more than 50 million are wasted (UNICEF, 2018).

Stunting rates are highest in several sub-Saharan Africa and south Asian countries, where the prevalence often exceeds 30%. In young children, stunting is associated with reduced physical and cognitive development, increased risk of infection, lower school achievement, and greater behavioral problems. Adults who were malnourished in childhood have less economic productivity, poorer maternal reproductive outcomes, and increased incidence of hypertension and glucose intolerance (UNICEF, 2018). Indeed, World Bank researchers reported that childhood stunting reduces the gross domestic product of affected countries by about 7% on average and by 10% for African and Asian countries, with the reduction being as high as 16% for certain countries (Galasso et al., 2016).

Stunting abounds among the poor in low-income settings where diets are cereal-based and lack diversity. The limited gastric capacity of infants, particularly infants, makes it difficult for them to ingest adequate nutrients needed to support rapid growth. Stunting is often associated with micronutrient deficiencies. For instance, 38% of children in India are stunted because young children mainly consume cereal-based food, which lacks easily digested protein and key bioavailable micronutrients (Shivakumar et al., 2019). These micronutrient deficiencies increase the risk of diseases such as diarrhea, malaria, and measles, further diminishing child growth and cognitive development. Micronutrient deficiencies in childhood are also associated with later reductions in work productivity, as well as poorer reproductive outcomes for women (Neumann et al., 2002).

### Importance of animal-sourced foods versus plant foods in meeting nutrient requirements

Compared with plant foods, animal-sourced foods provide dense and readily bioavailable sources of energy, protein, minerals, and vitamins. Animal-sourced foods are particularly valuable for infants in the first 1000 d of life when the small gastric size and rapid growth rate demand dense and bioavailable nutrient sources. The World Health Organization notes that animal-sourced foods are the best nutrient-dense foods for children aged 6 to 23 mo. Animal-derived proteins provide a balanced profile of amino acids that are readily digested, whereas plant-derived proteins often lack one or more amino acids critical for growth and other metabolic functions and are less digestible. For example, a recent study compared the digestibility of amino acids in rice, finger millet, mung dal, and eggs. The amino acid digestibility (measured by the digestible Indispensable Amino Acid Scores) was least for mung dal (65%), highest for eggs (87%), and intermediate for rice and finger millet (Shivakumar et al., 2019).

**Figure 2.**
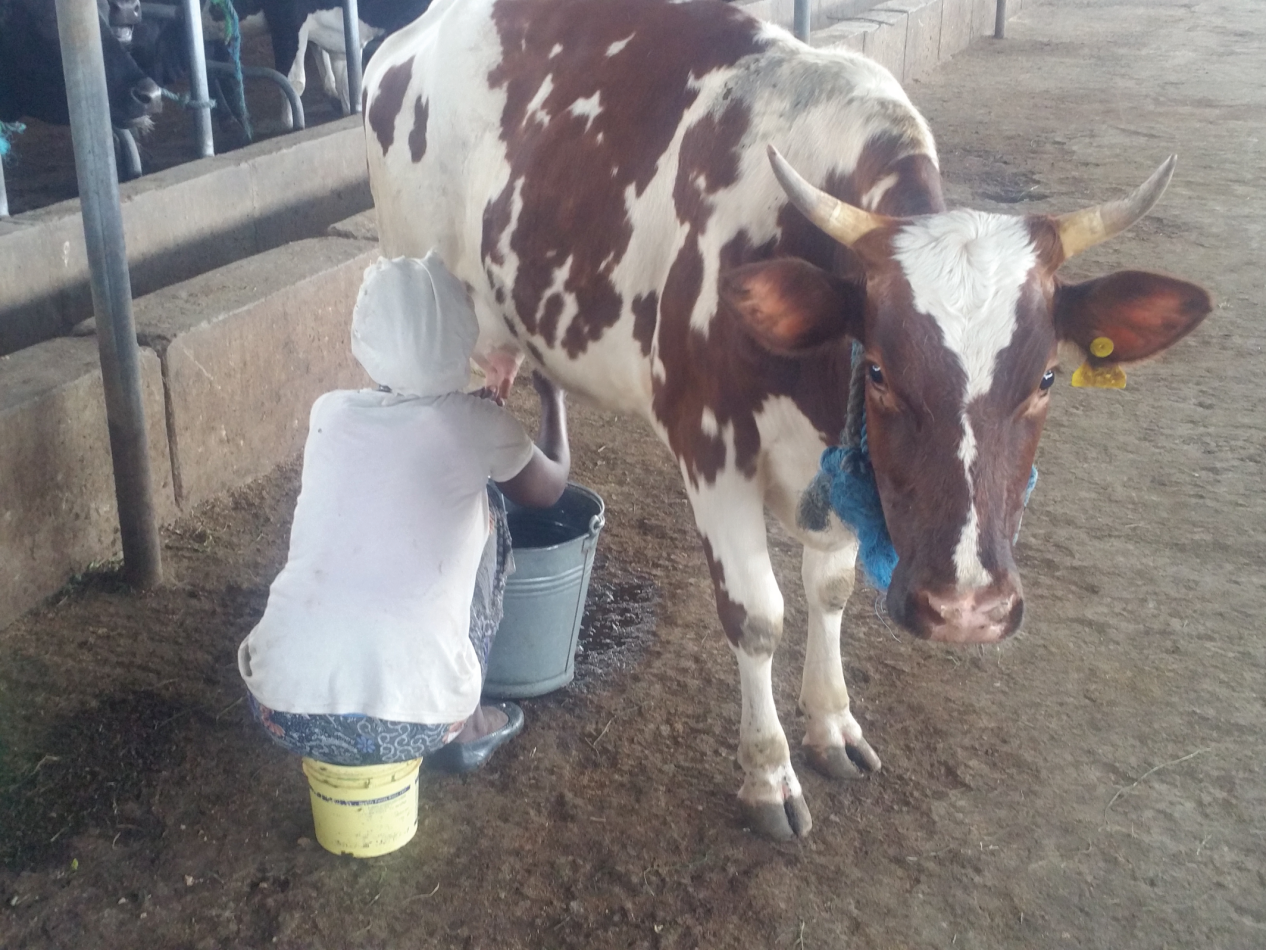
A woman milks a cow in Hawassa Ethiopia. Milk is a good source of essential vitmains and minerals such as vitamin A and calcium and is perhaps the best source of bioavailable iodine.

**Figure 3.**
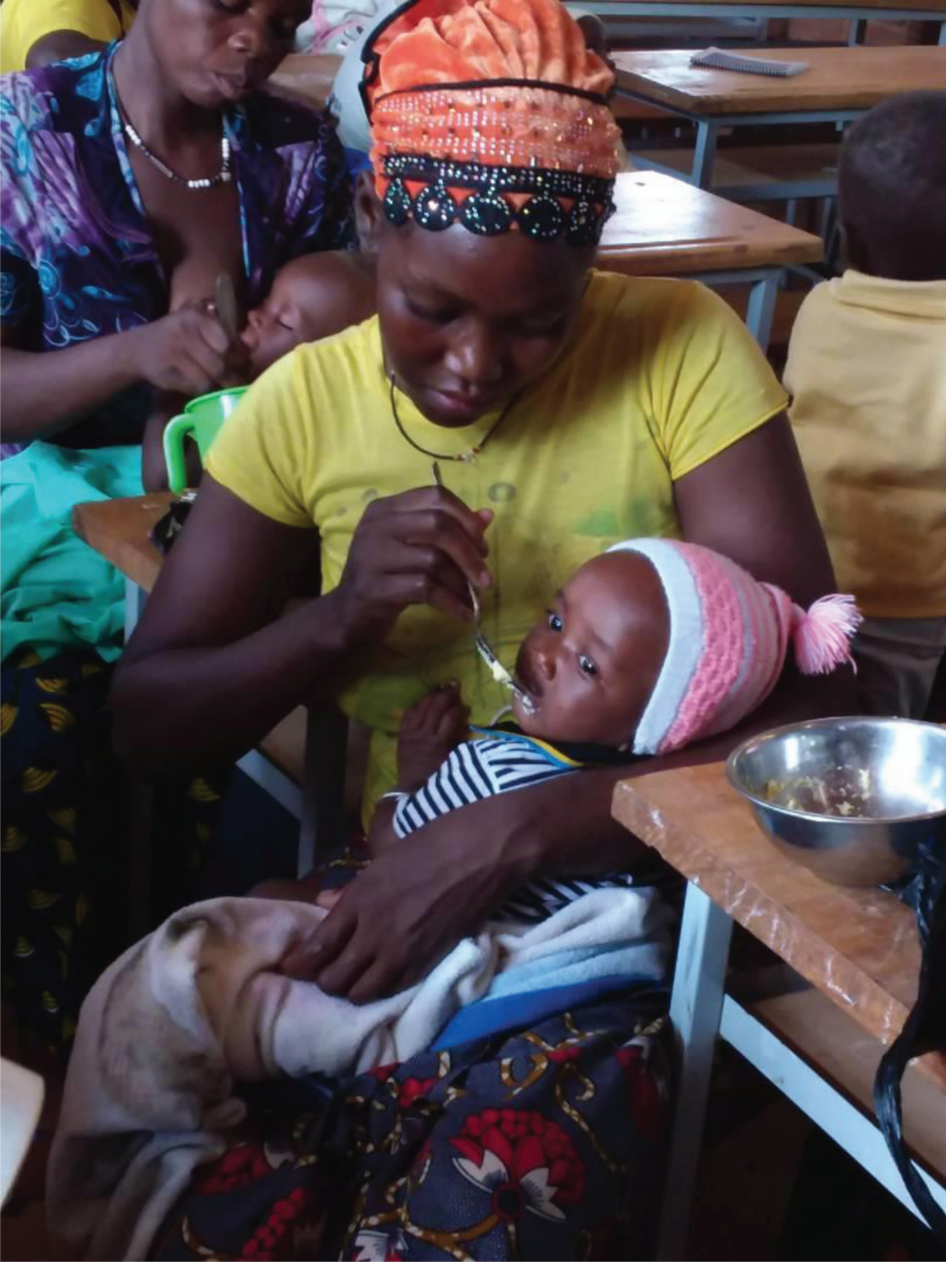
A woman feeds eggs to her baby in Burkina Faso. Eggs are good sources of the fatty acids docosahexaenoic acid (DHA) and eicosapentaenoic acid (EPA), which are important in brain development.

Dietary quality, rather than the quantity of food energy and protein, has been cited as a significant predictor of children’s cognitive development (Whaley et al., 2003). Intake of animal-sourced foods also improves growth, and physical activity of children, and leads to better pregnancy outcomes and reduced morbidity from illness (Neumann et al., 2002). Animal-sourced foods are important contributors to diet quality. For example, meat is rich in amino acids, iron, zinc, riboflavin, vitamin B12, vitamin B6, essential polyunsaturated fatty acids, and other micronutrients essential for cognitive function and normal growth. Milk (Figure 2) is a good source of vitamin A, calcium, vitamin B12, riboflavin, essential polyunsaturated fatty acids, folate (except goat’s milk which is folate deficient), and is perhaps the best source of bioavailable iodine. Eggs are good sources of amino acids (Figure 3), vitamins A, B2, B12, iodine, choline, folate, zinc, iron, and fatty acids such as docosahexaenoic acid (DHA) and eicosapentaenoic acid (EPA).

Therefore, animal-sourced foods provide many of the nutrients that are completely lacking (or less bioavailable) in plant-based foods.Animal-sourced foods also provide multiple micronutrients simultaneously. This can be important in the diets of the poor in low- and middle-income countries, which typically lack several nutrients. For example, about one-third of women globally are anemic; the prevalence is greatest in low- and middle-income countries (McLean et al., 2009). Vitamin A and riboflavin are both needed for iron mobilization and hemoglobin synthesis; therefore, iron supplementation or fortification alone may not successfully treat anemia if these other nutrients are deficient (Allen, 1995).

**Figure 4.**
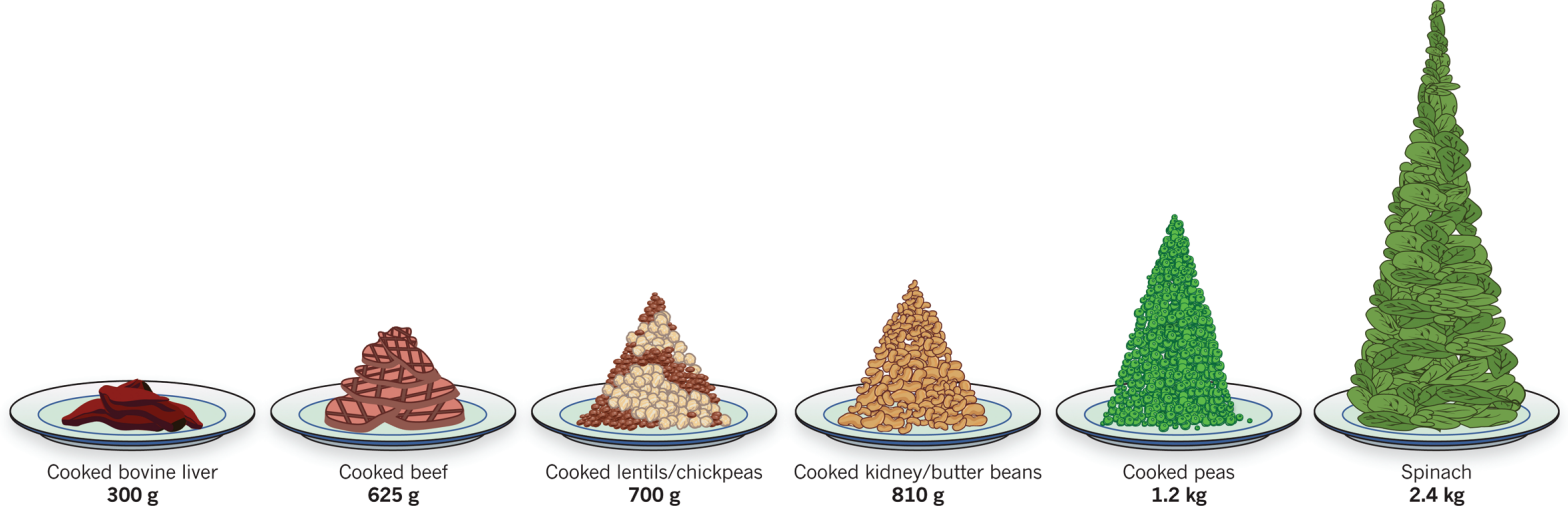
Amount of various types of foods that provide the same amount of iron. Figure provided by Gupta (2016).

**Figure 5.**
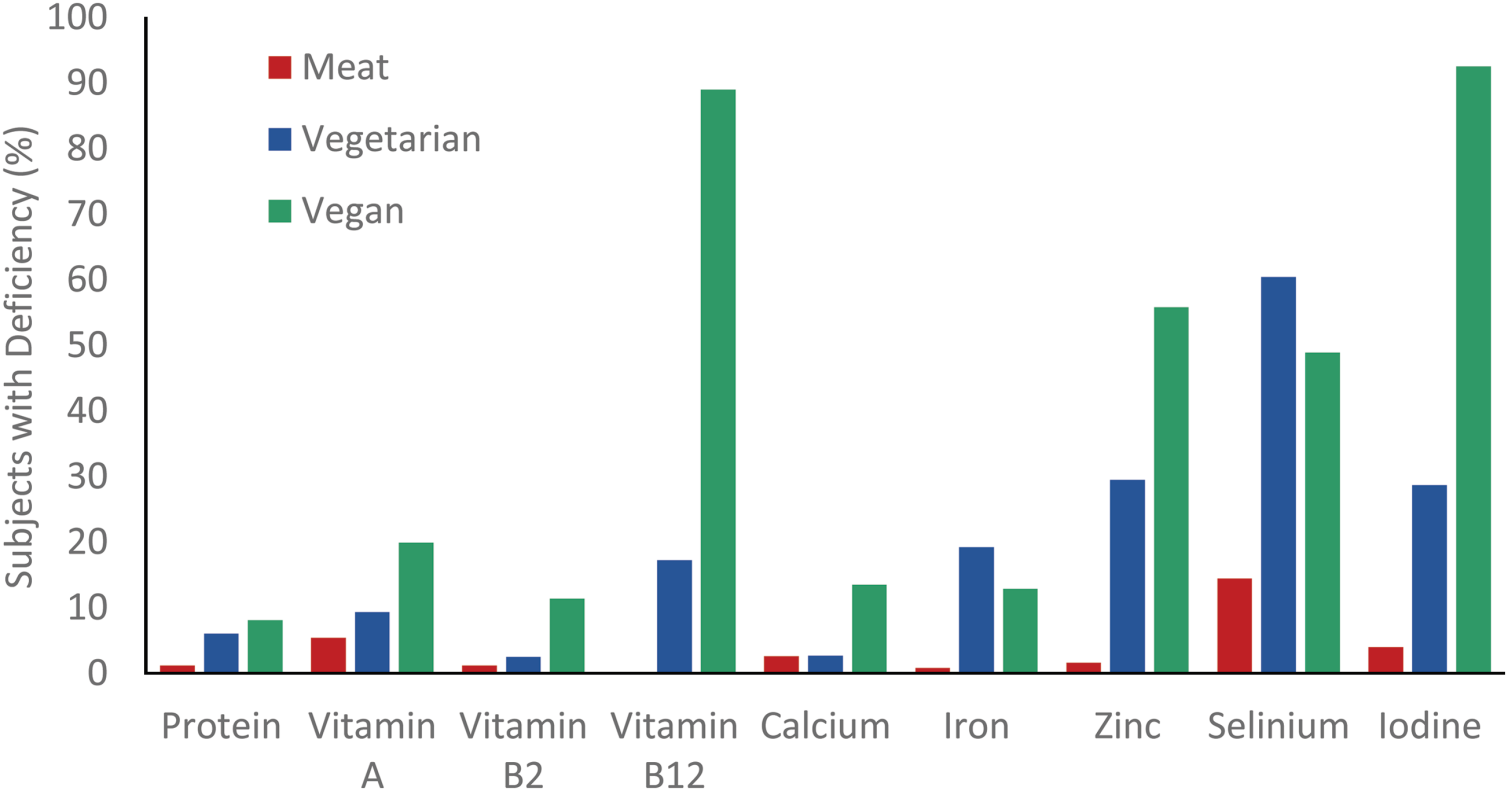
Incidence of common nutrient deficiencies among people consuming meat-, vegetarian-, or vegan-dominated diet patterns. Data are expressed as the percentage of subjects with deficiencies of protein, vitamins (A, B2, and B12), and minerals (calcium, iron, zinc, selenium, and iodine). Adapted from (Sobiecki et al., 2016; *n* = 24,000).

Consumption of even small amounts of animal-sourced foods contributes substantially to ensuring dietary quality. In fact, a woman would have to eat about 8 and over 3 times as much spinach as liver and beef to meet her daily iron needs, respectively (Gupta, 2016; Figure 4). Protein-energy malnutrition, iron-deficiency anemia, and vitamin A deficiency can be prevented if enough animal-sourced foods are included in the diet. This applies in low- and middle-income countries as well as developed countries; if animal-sourced foods are omitted from U.S. diets, micronutrient deficiencies will prevail (White and Hall, 2018). This is also evident from Figure 5 which compares nutrient deficiencies in meat eaters, vegetarians, and vegans in the United Kingdom (Sobiecki et al., 2016).

The foregoing clearly indicates that animal-sourced foods can significantly enhance nutritional quality and reduce malnutrition for vulnerable populations in low- and middle-income countries, especially young children and pregnant and lactating women. Animal-sourced foods are also important in meeting the nutrient needs of those in developed countries and moderate intakes may reduce the high rates of obesity and diabetes due to consumption of “empty” calories based on carbohydrate-dense foods in some of such countries.

### Evidence of the nutritional benefits of animal-sourced foods consumption

Research indicates that consumption of animal-sourced foods improves growth, cognition, and other nutrition outcomes in children. Consumption of various animal-sourced foods may affect these outcomes differently. For example, in some studies, milk was particularly associated with better linear growth and meat with better cognition (Neumann et al., 2007). Meat is a particularly good source of bioavailable iron, which is critically important for motor development and neurological functioning including learning and memory (Nyaradi et al., 2013). In a randomized controlled trial of dietary supplements for Kenyan school children, those whose diets were supplemented with meat outperformed children who received supplements of milk or oil (for energy) on cognitive performance and tests of arithmetic ability. The meat-supplemented group children had test scores 45% relative to baseline when their performance was averaged over five school semesters and all subjects, whereas those supplemented with milk, oil, and nothing (control) were 28% greater and 7% and 10% less, relative to baseline, respectively (Hulett et al., 2014). Iron-containing complementary foods like meat are especially important among infants who have insufficient iron stores or inadequate intake, as concluded in a recent systematic review (Obbagy et al., 2019).

There is increasing evidence on the importance of animal-sourced foods in reducing stunting. A meta-analysis (De Beer, 2012) showed that dairy consumption increased child growth, with a pooled effect increase in height of 0.4 cm per annum for additional consumption of 245 mL of milk daily. In Ecuador, adding one egg per day to the diet of young infants reduced stunting rates by nearly half (Iannotti et al., 2017). In India, adding an egg or milk to the diet reduced stunting in young children when such high-quality protein and micronutrient sources were consumed with a combination of cereals and legumes (Shivakumar et al., 2019).

Several other studies have reported improvement in various aspects of child development due to improved animal-sourced foods consumption. Examples include better motor, speech, and language development in Dutch children fed omnivore diets compared with those on vegetarian diets (Louwman et al., 2000); improvement in cognitive ability among school children in Kenya as a result of increased meat supplementation (Black, 2003); better pattern recognition memory in Ghanaian children supplemented with 8.8 g milk protein per day compared with those supplemented with 4.4 g milk protein per day (Lee et al., 2018); and greater head circumference (an indirect indicator of brain development and cognitive function) associated with animal-sourced foods consumption in Nepal (Miller et al., 2016). Despite all these positive findings, a recent systematic review concluded that there was only limited and low-quality evidence regarding the positive effects of animal-sourced foods on the growth and development of children aged 6 to 59 mo (Eaton et al., 2019). This is because studies examining effects of animal-sourced foods consumption on such measures are few and fewer still were randomized controlled longitudinal studies that involved simultaneously comparing diets that contained and lacked animal-sourced foods. Clearly, more work needs to be done to elucidate this relationship.

## Health Benefits of Animal-Sourced Foods

In addition to beneficial effects on growth and development, animal-sourced foods provide micronutrients and other elements important for human health. Iron and zinc are important for optimal function of the immune system and iodine is essential for thyroid function. Therefore, animal-sourced foods that contain these nutrients can contribute to enhanced immune systems. Cow milk is a source of potassium, which can enhance vasodilation and reduce blood pressure in adults. Calcium in milk is also important for bone health, blood clotting and wound healing, maintaining normal blood pressure, and muscle contractions including heartbeat. Dairy consumption improves bone health during childhood and adolescence and reduces the risk of osteoporosis and type 2 diabetes (Rozenberg et al., 2016). Dairy consumption has also been associated with reduced blood pressure, arterial stiffness, cardiovascular diseases, rickets, and hip fracture (Fekete, 2016). However, a systematic literature review on the effects of milk and dairy product consumption on prostate cancer risk and mortality (Lopez–Plaza et al., 2019) concluded that although there are some data indicating that higher consumption of dairy products could increase the risk of prostate cancer, the evidence is not consistent. In addition, meta-analysis by Guo et al. (2017) was not conclusive about the health benefits of milk consumption, further indicating the need for more studies. Similar equivocations also exist on the health benefits of meat and other animal-sourced foods indicating the need for further large scale, controlled, and longitudinal research studies for developing food guidelines.

## Economic Impacts of Sustainable Livestock Systems

**Figure 6.**
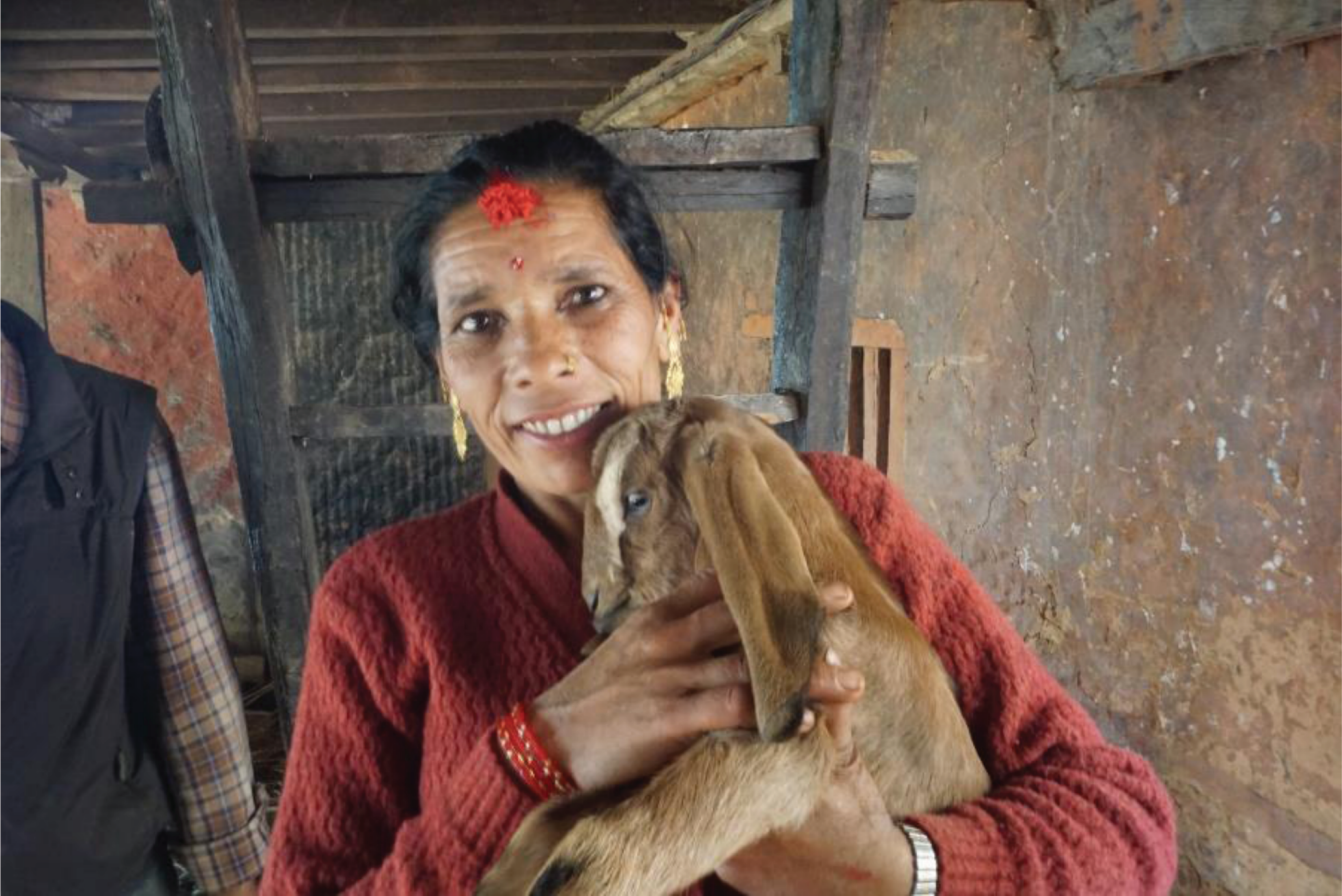
A Nepali woman proudly holds one of the goats from her flock. Livestock are a vital source of income and empowerment for women in low- and middle-income countries.

Livestock products (meat, milk, and eggs) are among the top 10 globally traded commodities with a value of approximately US$6.5 million (FAOSTAT, 2017). Livestock generate income for farmers of all categories via sale of animals and livestock products. In low- and middle-income countries, millions of farmers keep livestock as a status symbol, with more indicating greater status or as insurance against emergencies and sell them to meet cash needs; the animals are commonly referred to as a “savings bank on hooves” (Figure 6). Livestock also provide opportunities to capitalize on underutilized family labor. As the income from livestock is less seasonal (compared with crops), farmers, particularly women, depend on these animals as a vital source of income for household essentials, including payment of school fees and medical expenses. Livestock also serve to empower women who have important and varied roles in raising them in many low- and middle-income countries. The manure and draft power from livestock represent assets that can be used or sold as fuel for cooking or heating or building materials, or exchanged for needed commodities, respectively. Furthermore, income from livestock allows farmers to make better dietary and health choices and provide the necessary resources to pay for medical care.

According to the International Labor Organization, the livestock sector is an integral part of agriculture, which contributes 60% to 70% of total employment in low- and middle-income countries, mainly in Africa and Asia. The jobs in the sector are not limited to just farm production but extend to include aggregation, processing/value addition, distribution, transportation, food storage, retailing, food marketing, etc. Studies in Bangladesh and India have shown that raw milk collection and distribution creates 20 to 40 full time jobs per 1,000 liters of milk traded. Milk processing generates another 60 to 100 jobs per 1,000 liters of processed milk with around 15% of the traded milk being processed, leading to around 32 additional full-time jobs per 1,000 liters of marketed milk. It is to be noted that few comprehensive studies are currently available on the aggregate direct and indirect employment generation and socio-economic impacts of the livestock sector in low- and middle-income countries at the country or regional level.

## Differences in Animal-Sourced Foods Consumption Patterns and Underlying Causes

Various factors determine animal-sourced foods consumption patterns among different groups of people. In India, per capita consumption of milk was higher in urban than in rural areas (Kumar et al., 2014). This holds true in many countries. Not surprisingly, richer households consume significantly more milk and milk products than poor households (Kumar et al., 2014). Notably, an increase in purchasing power is associated with a change in the food consumption patterns; people include more meat, eggs, and milk products in their diets when their income increases. In other words, consumption of animal-sourced foods is income-elastic.

Consumption of milk is generally associated with ownership of dairy animals. A review of six studies conducted by the Global Dairy Platform to identify potential impacts of dairy farming revealed that ownership of dairy cattle resulted in a substantial increase in household milk consumption. However, when animal productivity is increased, it does not necessarily result in increased consumption of animal-sourced foods by the household members, especially in a market-oriented production scenario (Masset et al., 2011). It is difficult to ensure that the animal-keeping households increase their animal-sourced foods consumption when productivity of their animals increase. For example, several projects aiming to introduce or improve animal production suggest that livestock and their products are more likely to be sold for income than consumed by poor households (Ruel et al., 2018). Various sociocultural factors including religion and traditional beliefs also affect animal-sourced foods consumption and these are discussed below.

### Reasons for low/little consumption of animal-sourced foods

In several low- and middle-income countries, lack of access to animal-sourced foods is the main problem. This may be due to many reasons such as unavailability of animal-sourced foods at the right location, time, or form; lack of awareness about their importance in the diet; as well as poverty, gender dynamics, taboos, or other socio-cultural factors.

1) Awareness: Many of the poor who live in rural areas in low- and middle-income countries have little or no knowledge of nutrients and their importance to human health and well-being. Food consumption is mainly aimed at satisfying hunger; knowledge about the importance of animal-sourced foods in the diet is lacking.
2) Affordability/income: Compared with plant-based foods, animal-sourced foods are relatively expensive; thus, their consumption is income-dependent. Kumar et al. (2014) found that the per capita consumption of milk by rich households in India was 6.8 times higher than that of very poor households and 3.3 times higher than that of poor households. Similar trends are evident in other countries. In Ethiopia, the prices of dairy, eggs, and meat increased by about 30% over the last decade, whereas the price of grains, roots, and tubers did not increase (Bachewe et al., 2017). The relatively high cost of animal-sourced foods is a challenge for the poor who must make tough decisions on how to spend their scarce resources. Consequently, for many families, animal-sourced foods are not consumed at all, or only on rare occasions such as religious festivals.
3) Myths and taboos: Taboos associated with animal-source foods often create barriers to consumption of these foods. In Southern Ethiopia, consumption of animal-sourced foods by pregnant women is thought to be associated with a more difficult delivery (Demissie et al., 1998). Discussion with farmer/women groups in India and Nepal revealed that in some Indian communities it is believed that meat of scavenging poultry, buffalo, and pigs should not be consumed due to their dirty feeding habits. Some other communities believe that during menstruation, girls should not consume pure foods such as milk because they are impure. In Nepal, some people believe that milk is meant for consumption by evil spirits, and therefore, it should not be sold. In certain African countries, some people still believe that milk is for cats and not children, or that eating meat or eggs will make children steal, severely limiting animal-sourced food consumption.
a) Religion: Members of the Hindu faith avoid beef consumption due to veneration of cows. The caste system also limits animal-sourced foods consumption. For instance, Brahmins in India and Nepal do not eat beef and may not consume milk if the milking was done by someone in a low caste like the Dalits. It is also a belief that if the milk is consumed by lower caste people, the productivity of animals will be reduced (Mamgain and Diwakar, 2012). Muslims avoid pork consumption for religious reasons. In Ethiopia, devout orthodox Christians practice “fasting,” defined in this context as avoiding animal-sourced foods, for up to 240 d a year, during which adults and children eat food of suboptimal protein and calorie content.
b) Gender-based food allocation bias: There is ample evidence from all over the world to show that there is food allocation bias against females of all ages, and against younger household members. As a result, there are gender-based differences in the consumption of animal-sourced foods (Gittelsohn and Vastine, 2003). In South Asia, women, particularly pregnant, are discriminated against during allocation of food in households due to food insecurity or socio-cultural factors (Gittelsohn and Vastine, 2003). This is also true in many sub-Saharan African countries where the man gets the choice portions of the meal followed by the children. In many of such situations, women often eat last and the least; they fast more frequently and have limited decision-making power over food-purchasing decisions. Women also limit themselves from consuming enough animal-sourced foods for fear of big babies and the risk is thought to increase as women approach childbirth (Gittelsohn and Vastine, 2003).

### Pathways to promote consumption of animal-sourced foods among vulnerable groups

1) Education/training: Food choices are usually determined by availability, economic status, taste, convenience, social norms, etc., rather than nutritional knowledge. Therefore, creating awareness about healthy food options, especially during certain important stages of development (pregnancy, first 1000 d of life) is critical. Nutritional counseling and education can play a key role to promote good nutrition among the vulnerable in low- and middle-income countries. Proper education will also help us to address various taboos associated with consumption of animal-sourced foods. Community and religious leaders can assist in addressing cultural food allocation practices related to food type, gender, and age. Efforts are also needed to raise awareness among policy makers and researchers about the importance and benefits of animal-sourced foods consumption.
2) Increasing affordability: Since the high cost of animal-sourced foods is one of the main deterrents to their consumption, reducing their prices or improving the income of the poor would make them more accessible. The former can be achieved by increasing the efficiency and productivity of livestock, as well as the efficiency of actors along the livestock value chain (such as smallholder farmers, animal health workers, feed dealers, fodder producers, artificial insemination technicians, buck rearers, and marketing agents). This would increase the income of these groups of people. However, increased income and therefore increased affordability does not necessarily mean they will purchase and consume more animal-sourced foods. The households may choose other more expensive foods that do not supply the required nutrients. Therefore, nutritional interventions should include social behavioral change campaigns on the importance of animal-sourced foods in the diet.
3) Policies and programmes: As animal-sourced foods are relatively expensive, policies should be enacted and implemented to make livestock products more affordable or available for the poor. School lunch programs are one way this can be implemented. Similar efforts to improve the nutrition of school children in low- and middle-income countries with milk and eggs are being implemented by governments and nongovernmental organizations (NGOs) in various countries. Such efforts are commendable but inadequate. More nutrition-targeted subsidized programs focusing on children below the age of 5 yr, pregnant and lactating mothers, and people with low incomes, would help increase animal-sourced foods consumption at critical stages of growth. Certain corporations and multinational companies, particularly those dealing with livestock products (dairy, beef, and poultry industries), have established foundations and earmarked funds as part of their Corporate Social Responsibility. Similar initiatives and more of such programs are needed. Home-rearing of location-appropriate livestock species has also been promoted as a possibly pathway to improve household animal-sourced foods consumption as well as income.

### Sustainable livestock systems and the UN Sustainable Development Goals

**Figure 7.**
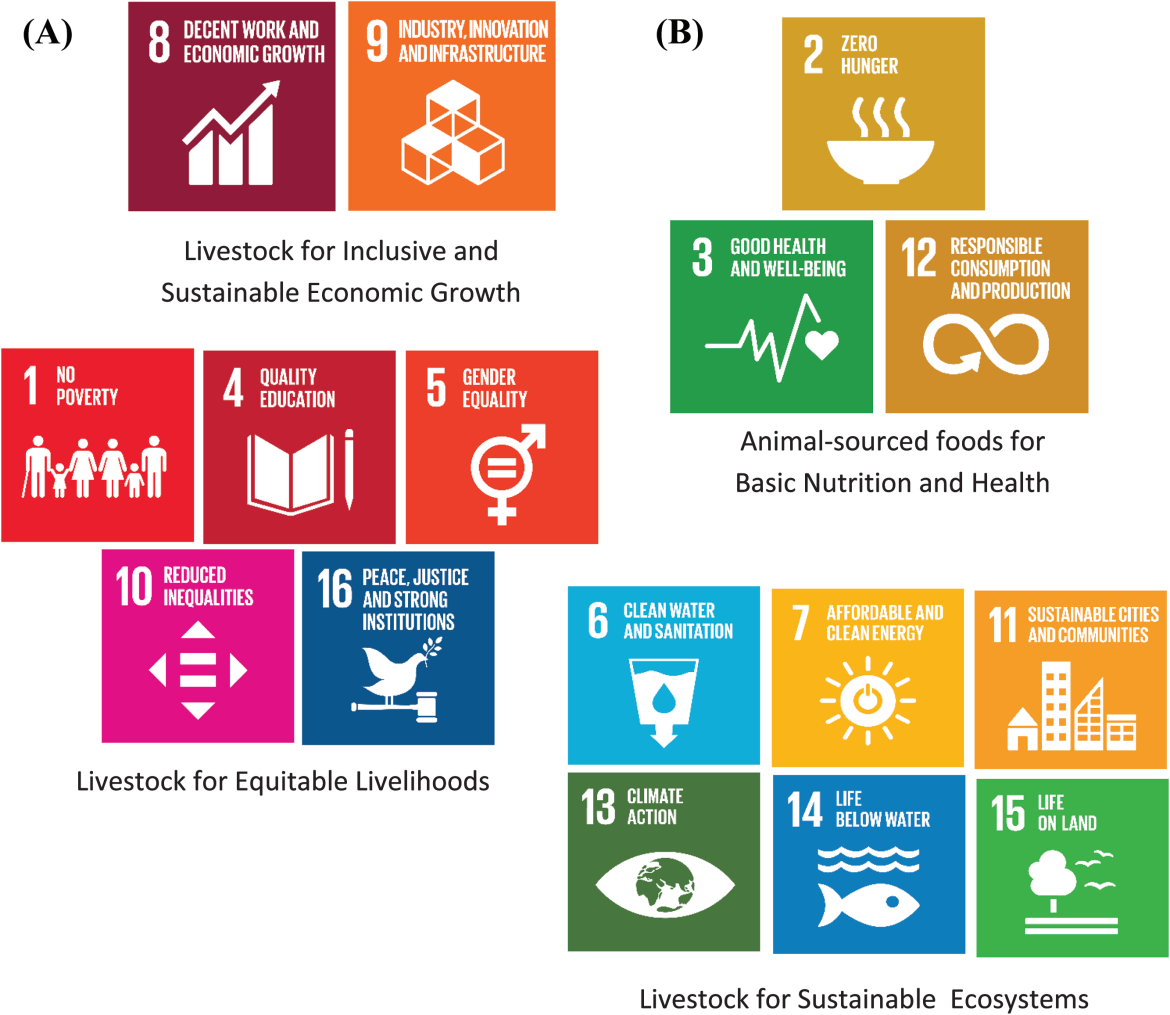
The role of livestock in achieving the United Nations sustainable development goals can be categorized into four main aspects including inclusive sustainable economic growth and equitable livelihoods (A) and improving nutrition and health, and sustainable ecosystems (B). Figure was adapted from Wright (2017).

The United Nations developed 17 Sustainable Development Goals (SDGs) as a blueprint to achieve a better and more sustainable future for all by 2030. The goals address global challenges including those related to poverty, inequality, climate, environmental degradation, prosperity, and peace and justice. Wright (2017) arranged the 17 SDGs into four groups (inclusive sustainable economic growth, equitable livelihoods, improving nutrition and health, and sustainable ecosystems) illustrating the critical roles livestock play in achieving the Sustainable Development Goals (Figure 7). These groupings and the associated descriptions of the role of livestock in each one indicate that achieving most of the SDGs without livestock is difficult and likely impossible.

## Conclusions

Livestock production contributes to environmental sustainability through conversion of human-unusable energy into highly nutritious animal-sourced foods, thereby contributing to the reduction in organic waste and pollution in the world, but also provide food and nutrition security. However, the potential and actual contribution of various livestock production systems to environmental sustainability varies according to production system. Various nutritional, genetic, management, and health-related strategies exist for reducing the environmental impact of livestock and making them contribute positively to sustainable livelihoods. Livestock contribute directly and indirectly to environmental and economic sustainability via various pathways. Some livestock systems are particularly effective at carbon sequestration and hence reducing greenhouse gas emissions that contribute to global warming. Assessment of the impact of livestock on the environment and livelihood should not focus on single criteria such as greenhouse gas emissions, but should balance ecological, social, and nutritional costs and benefits. Sustainable livestock systems contribute to food security, economic, environmental stewardship, and sociocultural needs and are vital for achieving most of the UN SDGs. They are particularly important for improving human nutrition, health, and economic productivity. Concerted efforts are needed to promote such systems in low- and middle-income countries.

## About the Authors

**Padmakumar Varijakshapanicker** has been working with International Livestock Research Institute (ILRI) since 2009 and is currently managing the Asia hub of the Livestock System Innovation Lab, a Feed the Future Initiative of USAID. He is also the Acting Head of ILRI’s NIRS Feed Technology Platform at Patancheru, Hyderabad, India. Padmakumar graduated from the Centre for Tropical Veterinary Medicine, University of Edinburgh, UK. He has been working in the livestock sector for more than three decades and has research experience in India, Nepal, and Cambodia.

**Adgebola (Gbola) Adesogan** is Director, Feed the Future Innovation Lab for Livestock Systems and Professor, Ruminant Nutrition at the University of Florida. His research interests include using animal-sourced foods to improve human health and nutrition; improving food and feed safety and quality; and devising sustainable strategies to optimize the performance, health, and welfare of livestock. Prior to his tenure at the University of Florida, he was an Assistant Professor of Animal Nutrition at the University of Wales, UK.

**Sarah L. McKune** is an Assistant Professor in the Department of Environmental and Global Health and the Center for African Studies at the University of Florida. She holds a BA in French and Sociology, a Master’s in Public Health, a PhD in Interdisciplinary Ecology, and was a postdoctoral fellow for the CGIAR’s research program on Climate Change, Agriculture, and Food Security. She currently serves as the Human Health and Nutrition Cross Cutting Theme leader for the Feed the Future Innovation Lab for Livestock Systems. Her research seeks to explain the complex household dynamics that affect child growth and nutritional outcomes, including behavior, women’s empowerment and gender dynamics, climate change, hygiene and sanitation, and livestock ownership.

**Laurie C. Miller** is Professor of Pediatrics, Adjunct Professor of Nutrition and of Child Development, Tufts University (Boston, USA). She works with Heifer Nepal as an investigator in several projects supported by the USAID Nutrition Innovation Lab and the Innovation Lab for Livestock Systems. She also serves as a consultant on zoonotic diseases to a World Bank-funded project in Nepal. She is currently a visiting professor and researcher in the Department of Child Psychiatry at Ste. Anne’s Hospital in Paris.

**Mulubrhan Balehegn Gebremikael** is an Associate Professor of Livestock Production and Pastoralist Development at Mekelle University, Ethiopia. He holds a PhD in Animal nutrition from the Norwegian Institute of Life Sciences and an MSc in Livestock Production and Pastoralist Development from Mekelle University. His research interests include improving the quality and productivity of animal feeds toward improving the productivity and consumption of animal-sourced foods in developing countries. He is currently working as a research coordinator at the University of Florida. Before his current position, Dr. Mulubrhan was a postdoctoral fellow at the United Nations International Ecosystem Management Partnership and at the Research and Publication Office, Mekelle University, Ethiopia.

**Saskia Hendrickx** is the Deputy Director of the Feed the Future Innovation Lab for Livestock Systems at the University of Florida. Saskia is a veterinary epidemiologist by training with over 15 yr of experience in public health, livestock research, and project management in developing countries. Before joining the University of Florida in 2016, Saskia worked with the International Livestock Research Institute and the World Health Organization. Her research aims to improve surveillance of zoonotic diseases and improve livestock production to increase household income in sub-Saharan Africa and Asia.

**Geoffrey E. Dahl** is the *Harriet B. Weeks* Professor in the Department of Animal Sciences at the University of Florida, Gainesville. Geoff conducts applied and basic research with direct impact on dairy production, including effects of photoperiod manipulation on production and health, the impact of frequent milking in early lactation on milk production, and heat stress abatement during the dry period on cow and calf productivity and health. He has active Extension programs in Sri Lanka, Nepal, Rwanda, and Ethiopia. Geoff is Past-President of the American Dairy Science Association.
