# Corrigendum to: Sustainable livestock systems to improve human health, nutrition, and economic status

**DOI:** 10.1093/af/vfz043

**Published:** 2019-12-02

**Authors:** Padmakumar Varijakshapanicker, Sarah Mckune, Laurie Miller, Saskia Hendrickx, Mulubrhan Balehegn, Geoffrey E Dahl, Adegbola T Adesogan

**Affiliations:** International Livestock Research Institute, Hyderabad, India; Department for Public Health and Health Professions, University of Florida, Gainesville, FL; Feed the Future Innovation Lab for Livestock Systems, Institute of Food and Agricultural Sciences, University of Florida, Gainesville, FL; School of Medicine, Friedman School of Nutrition Science and Policy, and Eliot-Pearson Department of Child Study and Human Development, Tufts University, Boston, MA; Feed the Future Innovation Lab for Livestock Systems, Institute of Food and Agricultural Sciences, University of Florida, Gainesville, FL; Department of Animal Sciences, Institute of Food and Agricultural Sciences, University of Florida, Gainesville, FL; Feed the Future Innovation Lab for Livestock Systems, Institute of Food and Agricultural Sciences, University of Florida, Gainesville, FL; Department of Animal, Rangeland and Wildlife Sciences, Mekelle University, Mekelle, Tigray, Ethiopia; Feed the Future Innovation Lab for Livestock Systems, Institute of Food and Agricultural Sciences, University of Florida, Gainesville, FL; Department of Animal Sciences, Institute of Food and Agricultural Sciences, University of Florida, Gainesville, FL; Feed the Future Innovation Lab for Livestock Systems, Institute of Food and Agricultural Sciences, University of Florida, Gainesville, FL; Department of Animal Sciences, Institute of Food and Agricultural Sciences, University of Florida, Gainesville, FL

There was an error in the first sentence of the section “Economic impacts of sustainable livestock systems” (page 44). The sentence, “Livestock products (meat, milk, and eggs) are among the top 10 globally traded commodities with a value of approximately US$6.5 million” has been corrected to reflect that the value is “approximately US$650 million”.

